# Direct targeting of wild-type glucocerebrosidase by antipsychotic quetiapine improves pathogenic phenotypes in Parkinson’s disease models

**DOI:** 10.1172/jci.insight.148649

**Published:** 2021-10-08

**Authors:** Lena F. Burbulla, Jianbin Zheng, Pingping Song, Weilan Jiang, Michaela E. Johnson, Patrik Brundin, Dimitri Krainc

**Affiliations:** 1 Department of Neurology, Northwestern University Feinberg School of Medicine, Chicago, Illinois, USA.; 2 German Center for Neurodegenerative Diseases (DZNE), Munich, Germany.; 3 Metabolic Biochemistry, Biomedical Center (BMC), Faculty of Medicine, Ludwig Maximilians University, Munich, Germany.; 4 Munich Cluster for Systems Neurology (SyNergy), Munich, Germany.; 5 Department of Chemistry, Chemistry of Life Processes Institute, Center for Molecular Innovation and Drug Discovery, and Center for Developmental Therapeutics, Northwestern University, Evanston, Illinois, USA.; 6 Parkinson’s Disease Center, Department of Neurodegenerative Science, Van Andel Institute, Grand Rapids, Michigan, USA.

**Keywords:** Neuroscience, Drug screens, Neurodegeneration, Parkinson disease

## Abstract

Current treatments for Parkinson’s disease (PD) provide only symptomatic relief, with no disease-modifying therapies identified to date. Repurposing FDA-approved drugs to treat PD could significantly shorten the time needed for and reduce the costs of drug development compared with conventional approaches. We developed an efficient strategy to screen for modulators of β-glucocerebrosidase (GCase), a lysosomal enzyme that exhibits decreased activity in patients with PD, leading to accumulation of the substrate glucosylceramide and oxidized dopamine and α-synuclein, which contribute to PD pathogenesis. Using a GCase fluorescent probe and affinity-based fluorescence polarization assay, we screened 1280 structurally diverse, bioactive, and cell-permeable FDA-approved drugs and found that the antipsychotic quetiapine bound GCase with high affinity. Moreover, quetiapine treatment of induced pluripotent stem cell–derived (iPSC-derived) dopaminergic neurons from patients carrying mutations in *GBA1* or LRRK2 led to increased wild-type GCase protein levels and activity and partially lowered accumulation of oxidized dopamine, glucosylceramide, and α-synuclein. Similarly, quetiapine led to activation of wild-type GCase and reduction of α-synuclein in a GBA mutant mouse model (*Gba1^D409V/+^* mice). Together, these results suggest that repurposing quetiapine as a modulator of GCase may be beneficial for patients with PD exhibiting decreased GCase activity.

## Introduction

Drug repurposing has the potential to generate urgently needed disease-modifying treatment for Parkinson’s disease (PD), the second most common neurodegenerative disease (1), more quickly and in a more cost-effective manner compared with traditional de novo drug discovery. PD is pathologically defined by the presence of intracellular proteinaceous inclusions, called Lewy bodies, primarily composed of insoluble aggregates of α-synuclein and the loss of dopaminergic neurons in the substantia nigra (2, 3). Variants in the *GBA1* gene encoding the lysosomal enzyme β-glucocerebrosidase (GCase) represent the most common genetic risk factor for PD (4). Loss-of-function *GBA1* mutations lead to the accumulation of its substrate glucosylceramide (GluCer) and α-synuclein, contributing to a toxic pathogenic cascade (5). We and others have previously shown that decreased wild-type GCase activity might also contribute to the pathogenesis in genetic and sporadic PD forms that are not linked to *GBA1* mutations (6–12). This suggests that activating the wild-type enzyme might be a viable therapeutic approach. We recently showed that modulation of wild-type GCase with a novel small molecule increased the enzyme activity and partially ameliorated pathological phenotypes in dopaminergic neurons from patients with *GBA1*-linked and non–*GBA1*-linked PD (13).

In this work, we used a fluorescent probe based on the modification of a GCase small molecule analogue (14) and screened FDA-approved drugs for their capacity to bind GCase in a fluorescence polarization (FP) high-throughput screen (HTS). We identified quetiapine, an antipsychotic commonly used to treat patients with dementia and psychosis (15), as the CNS drug with the greatest binding affinity. Moreover, we found that quetiapine increased wild-type GCase protein levels and activity and partially ameliorated dopamine oxidation and α-synuclein accumulation in iPSC-derived dopaminergic neurons from patients with *GBA1*-linked and LRRK2-linked PD. Administration of quetiapine to GBA mutant mice led to activation of GCase in the CNS and reduced α-synuclein accumulation. These results raise the possibility that quetiapine may modulate GCase activity and thereby slow disease progression in PD.

## Results

### Affinity-based FP screen of FDA-approved drugs identifies quetiapine as a binder of GCase.

**Figure 1:**
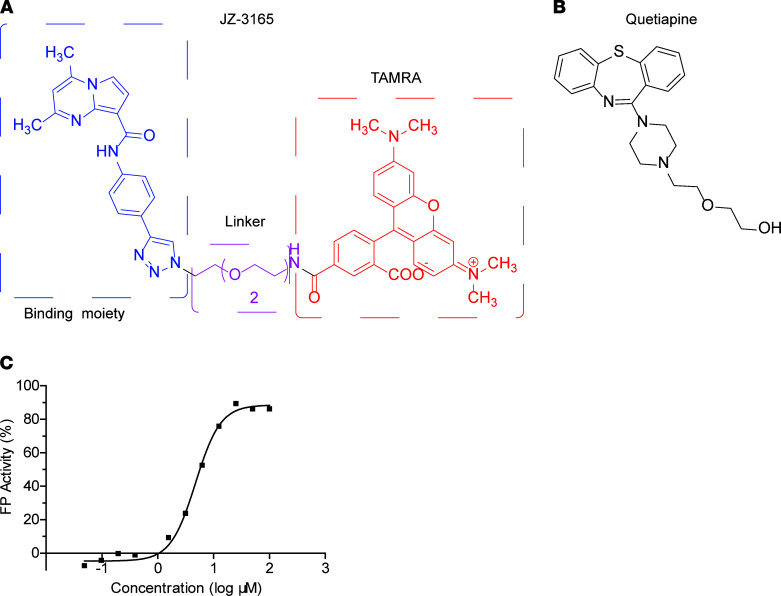
Affinity-based fluorescence polarization screen of FDA-approved drugs identifies quetiapine as a GCase binder. (**A** and **B**) Chemical structures of (**A**) fluorescence polarization (FP) probe JZ-3165 and (**B**) quetiapine. (**C**) Quetiapine FP activity in the FP assay using probe JZ-3165.

FP is a powerful technique for the nondisruptive measuring of biomolecular interactions in solution. One important application of FP-based assays is the FP competition assay to screen for small-molecule compounds that compete FP probe-protein interactions (16, 17). The principle of this assay relies on an increase of FP response once the fluorescent small-molecule probe is noncovalently bound to the protein of interest. Equilibrium displacement of the protein-bound probe by a test compound results in reduced FP due to the faster rotation of the unbound probe (16). To facilitate the discovery of noninhibitory GCase modulators, we designed the FP probe, JZ-3165 (Figure 1A), by linking a fluorescent probe (TAMRA) to an optimized binding moiety. The latter was based on a structure-activity relationship study of the GCase compound with a short polyethylene glycol linker. JZ-3165 exhibited a dissociation constant (*K_D_*) value of 0.71 μM binding affinity with recombinant GCase. Using this probe, we developed an affinity-based FP HTS to screen 1280 structurally diverse, bioactive, and cell-permeable compounds approved by the FDA. We identified a group of drugs that were able to bind GCase with varying affinity (Supplemental Table 1; supplemental material available online with this article; https://doi.org/10.1172/jci.insight.148649DS1), of which the CNS drug quetiapine (Figure 1B) showed the greatest affinity, with a *K_D_* value of 5 μM (Figure 1C). Among the identified hits, perphenazine and fluphenazine had similar chemical structures as quetiapine, indicating that they may share the same binding site on GCase, but they exhibited higher *K_D_* values, making them less likely to affect GCase levels and activity.

### Quetiapine improves pathogenic phenotypes in iPSC-derived dopaminergic neurons from patients with GBA1-linked PD.

**Figure 2:**
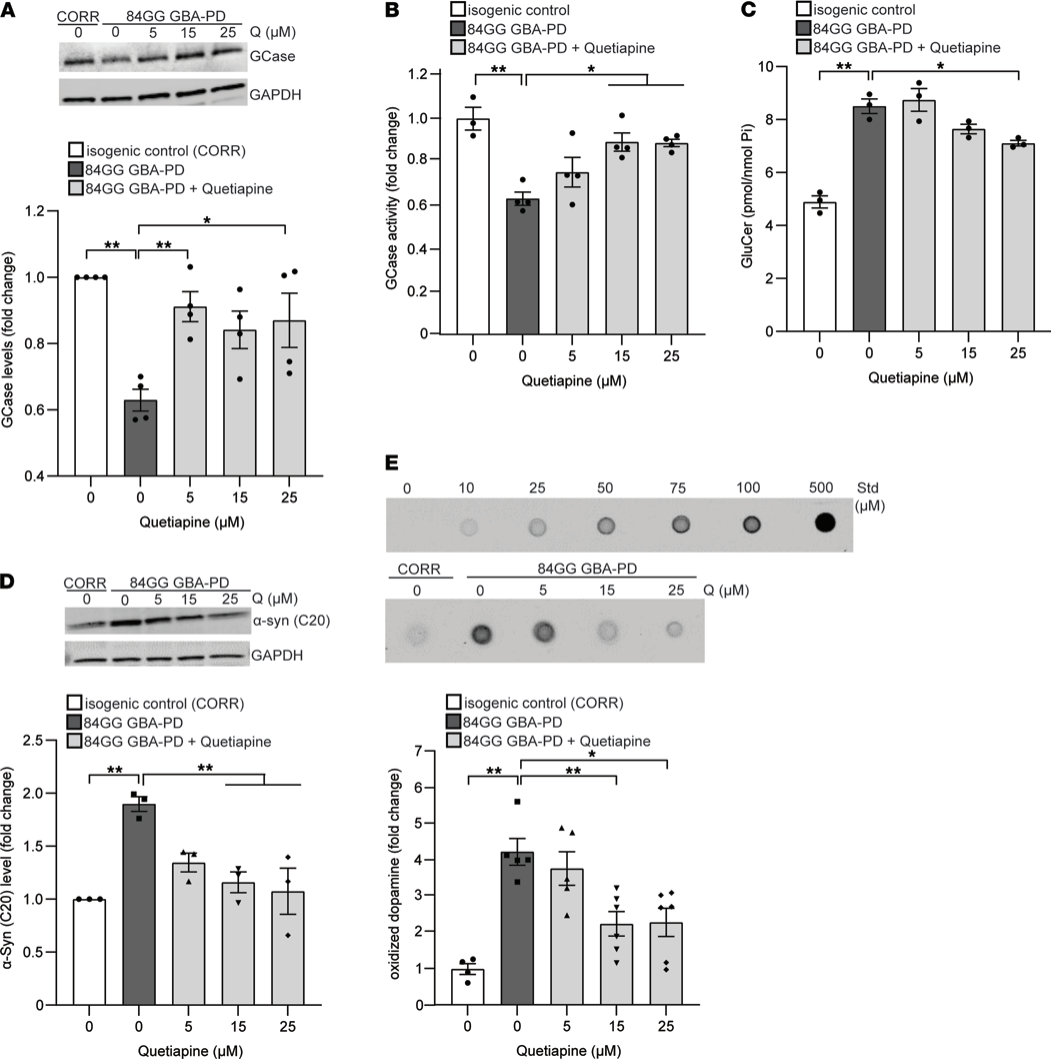
Quetiapine increases wild-type β-glucocerebrosidase and lowers pathogenic phenotypes in *GBA1*-linked iPSC-derived dopaminergic neurons. (**A** and **B**) Heterozygous 84GG *GBA1* mutant dopaminergic neurons (84GG GBA-PD) and isogenic control neurons with *GBA1* mutation corrected by CRISPR/Cas9 gene editing (CORR) were treated with DMSO (vehicle) or quetiapine (5, 15, and 25 μM) for 10 days. All samples were collected at day 130 of differentiation. Triton-soluble lysates were analyzed for (**A**) GCase protein by immunoblotting (*n* = 4 independent experiments) and (**B**) GCase activity by in vitro enzyme activity assay (*n* = 3–4 independent experiments). (**C**) Quantification of intracellular total glucosylceramide (GluCer) species by mass spectrometry normalized to internal phosphate (Pi) (*n* = 3 independent experiments). (**D**) Immunoblot analysis of α-synuclein in Triton-soluble lysates (*n* = 3 independent experiments). (**E**) Detection and quantification of oxidized dopamine (DA) performed by near-infrared fluorescence assay (*n* = 4–6 independent experiments). Standard of oxidized DA ranging from 0 to 500 μM is shown. Error bars represent mean ± SEM. *P < 0.05, **P < 0.01, 1-way ANOVA with Tukey’s post hoc test. Std, standard; Q, quetiapine.

To determine whether quetiapine could modulate wild-type GCase levels and activity in human neurons, we examined iPSC-derived dopaminergic neurons from a PD patient line harboring the heterozygous 84GG *GBA1* mutation (c.84dupG frame-shift mutation) that results in complete loss of mutant GCase and reduced wild-type protein from a single copy of the wild-type GCase allele and used a CRISPR-corrected isogenic line as control. We found reduced GCase protein levels and activity in the *GBA1* patient line compared with those in an isogenic control line, which were partially corrected by treatment with increasing concentrations of quetiapine (Figure 2, A and B). Next, we examined whether increasing wild-type GCase levels and activity were sufficient to modify disease-related pathogenic phenotypes in patient neurons. Treatment with quetiapine partially reduced the accumulation of intracellular total GluCer species (Figure 2C) and lowered α-synuclein levels in the Triton-soluble fraction (Figure 2D), whereas levels of glucosylsphingosine and Triton-insoluble α-synuclein were not changed (Supplemental Figure 1). We have recently reported that dopaminergic neurons from patients with familial or sporadic PD exhibit accumulation of oxidized dopamine that contributes to lysosomal dysfunction (6, 13). Interestingly, increasing concentrations of quetiapine partially ameliorated accumulation of oxidized dopamine in neurons from patients with *GBA1* PD compared with isogenic control neurons (Figure 2E and Supplemental Figure 2).

### Quetiapine treatment leads to partial rescue of pathogenic phenotypes in LRRK2-linked PD iPSC-derived dopaminergic neurons.

**Figure 3:**
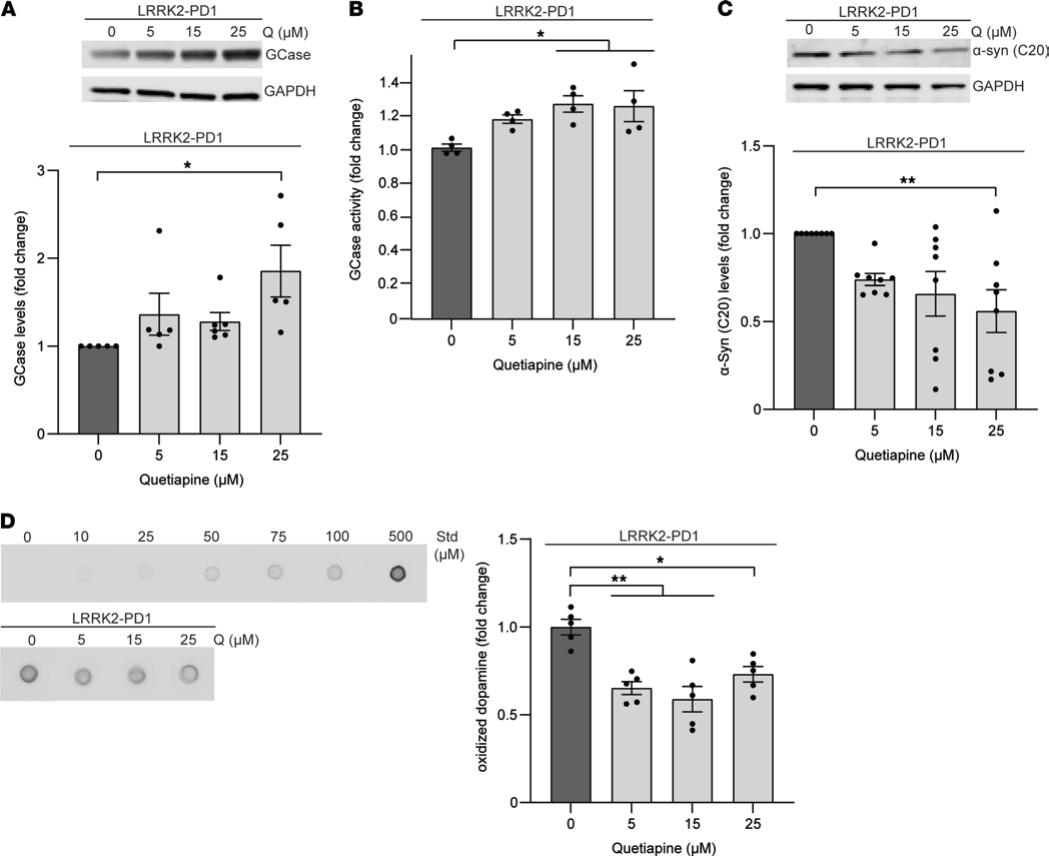
Quetiapine treatment leads to wild-type β-glucocerebrosidase activation and partial rescue of pathogenic phenotypes in non–*GBA1*-linked PD iPSC-derived dopaminergic neurons. LRRK2 G2019S mutant dopaminergic neurons (LRRK2-PD1) were treated with DMSO (vehicle) or quetiapine (5, 15, and 25 μM) for 10 days. All samples were collected at day 100 of differentiation. (**A** and **B**) Triton-soluble lysates were analyzed for (**A**) GCase protein by immunoblotting (*n* = 5–6 independent experiments) and (**B**) GCase activity by in vitro enzyme activity assay (*n* = 4 independent experiments). (**C**) Immunoblot analysis of α-synuclein in Triton-soluble lysates (*n* = 8 independent experiments). (**D**) Cell lysates were analyzed for oxidized dopamine (DA) by near-infrared fluorescence assay (*n* = 5 independent experiments). Standard of oxidized DA ranging from 0 to 500 μM is shown. Error bars represent mean ± SEM. *P < 0.05, **P < 0.01, 1-way ANOVA with Tukey’s post hoc test. Std, standard; Q, quetiapine.

Previous reports have shown that wild-type GCase activity is reduced in brain tissue and iPSC-derived neurons from patients with genetic and sporadic forms of PD (6–11), suggesting that improving wild-type GCase activity may prove beneficial. Here, we sought to investigate whether treatment with quetiapine would be sufficient to increase GCase levels and activity in mutant LRRK2 G2019S PD patient lines that exhibit decreased activity of wild-type GCase (18). Treatment with quetiapine led to an increase in GCase protein levels (Figure 3A) as well as enzyme activity (Figure 3B) and partially reduced the accumulation of α-synuclein in the Triton-soluble (Figure 3C) as well as Triton-insoluble fraction (Supplemental Figure 3) in neurons from patients with LRRK2 PD (LRRK2-PD1). We previously found accumulation of oxidized dopamine in dopaminergic neurons from patients with various forms of non–*GBA1*-linked PD, including LRRK2 (6, 11, 13). Importantly, treatment of neurons from patients with LRRK2 PD with quetiapine led to decreased levels of toxic oxidized dopamine (Figure 3D). These findings were confirmed in another LRRK2 G2019S PD patient line (LRRK2-PD2), whereby an increase of GCase activity upon quetiapine treatment resulted in the reduction of oxidized DA (Supplemental Figure 4).

### Wild-type GCase activation by quetiapine treatment in mice lowers α-synuclein accumulation.

**Figure 4:**
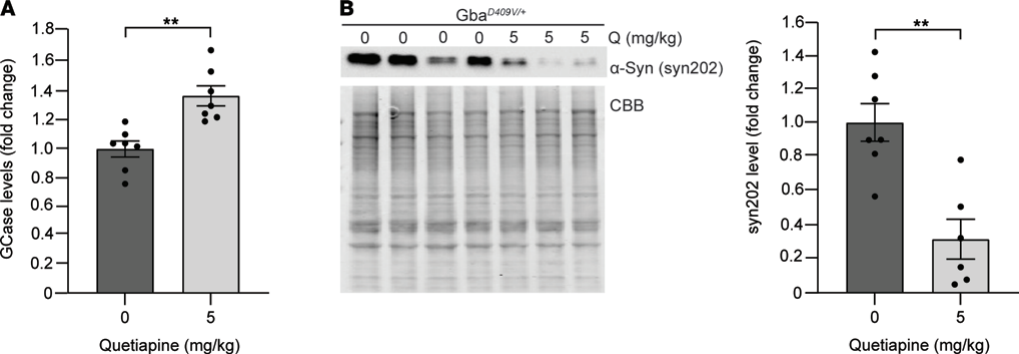
Wild-type β-glucocerebrosidase activation by quetiapine treatment in mice lowers α-synuclein accumulation. *Gba1^D409V/+^* mutant mice were treated with saline (vehicle) or quetiapine (5 mg/kg) intraperitoneally twice daily for 15 days. (**A**) β-Glucocerebrosidase (GCase) activity was measured by in vitro enzyme activity assay in Triton-soluble lysates of hippocampal tissue (*n* = 7 saline- and *n* = 7 quetiapine-treated mice) (**B**) Immunoblot analysis of α-synuclein in Triton-insoluble lysates of hippocampal tissue (*n* = 7 saline- and *n* = 6 quetiapine-treated mice). Coomassie Brilliant blue (CBB) was used as a loading control. Error bars represent mean ± SEM. **P < 0.01, Student’s *t* test. Q, quetiapine.

To determine whether quetiapine can modulate GCase activity in vivo, it was administered, via intraperitoneal injections, at 5 mg/kg to *Gba1^D409V/+^* mutant mice twice daily for 15 days. This led to an increase in GCase activity in brain tissue from mice treated with quetiapine compared with that in vehicle-treated mice (Figure 4A). Activation of GCase with quetiapine also reduced Triton-insoluble α-synuclein (19) in brain tissues of *Gba1^D409V/+^* mutant mice (Figure 4B), whereas Triton-soluble α-synuclein was not changed (Supplemental Figure 5). These results suggest that quetiapine can modify GCase activity in vivo and has beneficial effects on reducing α-synuclein.

## Discussion

Our results suggest that increasing wild-type GCase activity could serve as a potential therapeutic approach for various forms of PD that exhibit decreased GCase activity. Decreased GCase activity contributes to PD pathogenesis not only in patients who carry *GBA1* mutations, but also in patients with sporadic or other genetic forms of PD who do not harbor *GBA1* mutations (6–12). We have previously described that GCase and α-synuclein form a positive feedback loop, in which α-synuclein inhibits trafficking of wild-type or mutant GCase to lysosomes, leading to decreased lysosomal GCase activity that in turn contributes to α-synuclein accumulation (5). In addition, we found that vulnerable midbrain neurons in multiple forms of PD accumulate toxic oxidized dopamine that modifies key cysteine residues in the catalytic site of wild-type GCase, further decreasing GCase activity (6). Previous screens for GCase modulators measured enzyme activity using artificial substrates that are not physiologically relevant, such as 4-methylumbelliferyl-β-D-glucopyranoside and resorufin β-D-glucopyranoside, and identified hit compounds were not validated with GluCer-like substrates (14). Using FP HTS and a unique probe, in this study we were able to identify quetiapine as a potentially novel GCase modulator that also ameliorates key pathogenic effects linked to deficient GCase activity in dopaminergic neurons. It will be of interest to examine the effects of quetiapine treatment in other genetic and sporadic forms of PD that exhibit decreased activity of wild-type GCase.

Quetiapine was developed in 1985, was approved for medical use in the United States in 1997, and is now a commonly prescribed CNS drug, primarily for treatment of bipolar disorder, depression, and schizophrenia (20). Quetiapine is both effective and well tolerated, with fewer side effects than other antipsychotics, especially in patients with dementia who also exhibit psychosis (21). Repurposing strategies of currently approved and previously studied drugs facilitate speeding up of the traditional process of drug discovery by identifying a clinical use for drugs that have already proved to be safe and effective in humans. This strategy, which is also actively explored in PD (22), may also lower overall costs and shorten development timelines (23). Our findings highlight the potential value of quetiapine as a therapeutic approach for different forms of PD, including familial and sporadic PD, which exhibit decreased wild-type GCase activity. Future clinical studies will be required to fully evaluate quetiapine’s potential for the treatment of PD and to define appropriate doses for long-term therapy.

## Methods

### Cell-free enzyme activity assay.

Cell-free in vitro enzyme activity assay was performed as described previously (24). The recombinant GCase enzyme velaglucerase alfa (Vpriv, Shire Human Genetic Therapies Inc.) was obtained from residual solution after clinical infusions.

### Determination of probe (JZ-3165) binding affinity by FP assay.

Synthesis of FP probe JZ-3165 has previously been reported (25). JZ-3165 (25 nL/well, 50 nM final concentration) was transferred into 384-well black plates using the Labcyte Echo 550 Liquid Handler system. 25 μL/well enzyme dilutions in GCase activity assay buffer (final titration: 5 nM to 10 μM, 10 concentrations, 2-fold dilution) were added. A buffer solution without GCase protein was used as a blank control. The plate was shaken at room temperature in the dark for 20 minutes. FP was measured using a Tecan Infinity M1000 microplate reader (Ex = 535 nm and Em = 58 0 nm).

### In vitro FP HTS using JZ-3165 as a probe.

A mixture of GCase protein (2 μM) and JZ-3165 probe (50 nM) in GCase enzyme activity buffer (2 μL/well) was added into a NUNC black 1536-well plate. A single dose (5 nL, 25 μM final concentration) of drugs in DMSO (10 mM) solution was transferred into the plate, incubated for 20 minutes, and read at established conditions. Activities were confirmed by dose-response curves using freshly prepared solutions from solid samples.

### Generation of human iPSCs and differentiation into midbrain dopaminergic neurons.

Both iPSC lines from PD patients with mutations in *GBA1* were obtained from Northwestern University Biorepository (13). The 2 iPSC lines from PD patients with G2019S LRRK2 mutations were obtained from the National Institute of Neurological Disorders and Stroke Repository at the Coriell Institute for Medical Research (catalog no. ND29423 and ND29492).

iPSCs were maintained in mTeSR1 (StemCell Technologies) on Matrigel-coated (Corning) plates. Dopaminergic neuron differentiation was performed according to published protocols (26) and as previously described (6). Neural growth factors were removed on day 40, and cells were maintained in Neurobasal Media (Thermo Fisher Scientific, 21103-049) with NeuroCult SM1 supplement (STEMCELL Technologies, 5711). All iPSC lines were found to differentiate at similar efficiencies. Results were obtained from a set of at least 3 independent dopaminergic neuron differentiations. All iPSC lines were routinely tested for mycoplasma contamination.

### Gba1^D409V/+^ mice.

*Gba1^D409V/+^* (C57BL/6N-GBAtm1.1Mjff) mice (The Jackson Laboratory) were bred following guidelines from the Institutional Animal Care and Use Committee at Northwestern University and handled in accordance with the US NIH *Guide for the Care and Use of Laboratory Animals* (National Academies Press, 2011) and Society for Neuroscience guidelines. Mice (mixed group of male and female animals) aged 17–19 months received saline or quetiapine (5 mg/kg) through intraperitoneal injection, twice per day, 5 days per week, over 3 weeks.

### Sequential biochemical extraction.

Sequential biochemical extraction of proteins from iPSC-derived neurons and mouse tissue has been previously described (6). Neurons were harvested and centrifuged at 300*g* for 5 minutes, and cell pellets were homogenized for extraction in 1% Triton X-100 lysis buffer. After incubation on ice for 30 minutes, samples were centrifuged at 100,000*g* for 30 minutes at 4°C. Supernatant was analyzed as the Triton-soluble fraction. Insoluble pellets were extracted in 2% SDS/50 mM Tris (pH 7.4) buffer and further centrifuged at 150,000*g* for 30 minutes at 4°C. SDS-insoluble pellets were further processed for near-infrared fluorescence assay. For biochemical extraction of brain tissue from mice, hippocampal brain tissue was homogenized in 1% Triton X-100 lysis buffer according to tissue weight. Insoluble pellets from a 100,000*g* spin were extracted in 2% SDS/50 mM Tris (pH 7.4) by boiling and sonication. Samples were centrifuged at 150,000*g* for 30 minutes at 4°C, and the supernatant was analyzed as the SDS-soluble fraction.

### Near-infrared fluorescence detection of oxidized dopamine.

The near-infrared fluorescence assay was performed as described previously (6, 27). Leftover pellets from SDS extraction were extracted in 1 M NaOH at 55°C overnight. Then, samples were dried, washed with H_2_O, lyophilized, and solubilized in H_2_O. Samples and standard solutions from a 10 mM oxidized dopamine stock were dropped on a Biodyne Nylon Transfer Membrane (Pall). Membranes were scanned using Odyssey infrared imaging system (LI-COR) with the 700 channel. Samples were quantified by obtaining integrated spot intensities using Odyssey infrared imaging software (LI-COR).

### Antibodies.

The following primary antibodies were used: rabbit anti-GCase (MilliporeSigma, G4171, 1:1000), α-synuclein (C-20, Santa Cruz Biotechnology, SC-7011-R, 1:1000), α-synuclein (syn202, BioLegend, MMS-529R, 1:1000), and GAPDH (MilliporeSigma, MAB374, 1:5000).

### Assay for in vitro activity of GCase in neurons and mouse brain tissue.

Activity was measured from Triton-soluble fractions of whole-cell neuronal lysates as described previously (10). Hippocampal brain tissue from mice was homogenized in 1% Triton X-100 lysis buffer according to tissue weight and processed and analyzed as previously described (13).

### Quantification of GluCer by SFC/MS/MS analysis.

Neurons were harvested in cold PBS and centrifuged at 200*g* for 5 minutes; cell pellets were rapidly stored at –80°C. Quantification of GluCer and glucosylsphingosine was performed as a service provided by the Lipidomics Core facility at the Medical University of South Carolina. Samples were normalized to total cellular phosphate (Pi) levels and expressed as picomole/nanomole Pi.

### Statistics.

Comparison of multiple groups was performed using 1-way ANOVA followed by Tukey’s post hoc test; comparisons between 2 groups were performed using 2-tailed *t* tests. *P* values of less than 0.05 were considered significant. All data shown are representative of experiments from *n* ≥ 3 independent experiments. All errors bars represent SEM.

### Study approval.

Animal studies were approved by the Northwestern University Animal Care and Use Committee.

## Author contributions

DK was responsible for the overall direction of the project. LFB was responsible for Figures 2 and 3; Supplemental Figure 1A; Supplemental Figure 2, C, D, F, and G; and Supplemental Figure 4. JZ was responsible for Figure 1. PS was responsible for Figure 4; Supplemental Figure 1B; Supplemental Figure 3; and Supplemental Figure 5. WJ was responsible for Supplemental Figure 2, A, B, and E. JZ developed, performed, and analyzed the results of the FP HTS. MEJ and PB provided expert advice and critical review of the manuscript. LFB constructed the figures and analyzed the results shown in Figures 2–4. LFB, JZ, and DK wrote the manuscript.

## Supplementary Material

Supplemental data

## Version 1 10/08/2021

Electronic publication
