# Supplementary material for: Direct targeting of wild-type glucocerebrosidase by antipsychotic quetiapine improves pathogenic phenotypes in Parkinson’s disease models
Source: JCI Insight. 2021 Oct 8;6(19):e148649. doi: 10.1172/jci.insight.148649 (PMC8525588; doi:10.1172/jci.insight.148649)
Supplement: Supplemental data [file jciinsight-6-148649-s064.pdf]

Supplementary Materials for

Direct targeting of wild-type glucocerebrosidase by antipsychotic quetiapine improves pathogenic phenotypes in models of Parkinson's disease

Lena F. Burbulla[1*], Jianbin Zheng[1,2*], Pingping Song[1], Weilan Jiang[1], Michaela E. Johnson[3], Patrik Brundin[3] and Dimitri Krainc[1#]

Correspondence to: dkrainc@nm.org

This PDF file includes:

Table S1

Fig. S1 – S5

Supplementary Table 1

Drugs	Structure	JZ-3165 FP (µM)	4-MU-β-Gly enzyme activity (µM)	Disease
Dasatinib	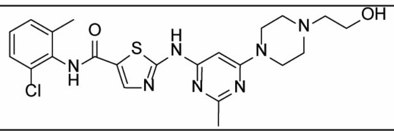	7.1	NA	Cancer
Cabozantinib	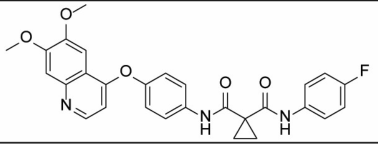	4.46	NA	Cancer
Vortioxetine	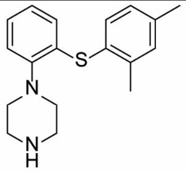	14.1	NA	CNS
Perphenazine	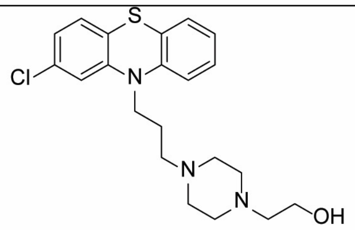	12.6	NA	CNS
Quetiapine	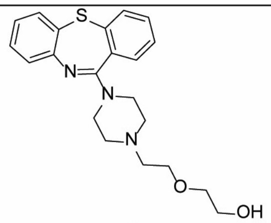	5.0	NA	CNS
Fluphenazine	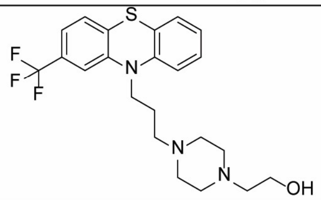	11.2	NA	CNS
Tegaserod	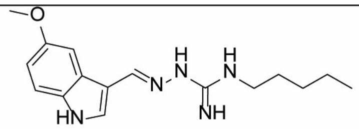	2	12.5, inhibitor	bowel syndrome

Figure S1

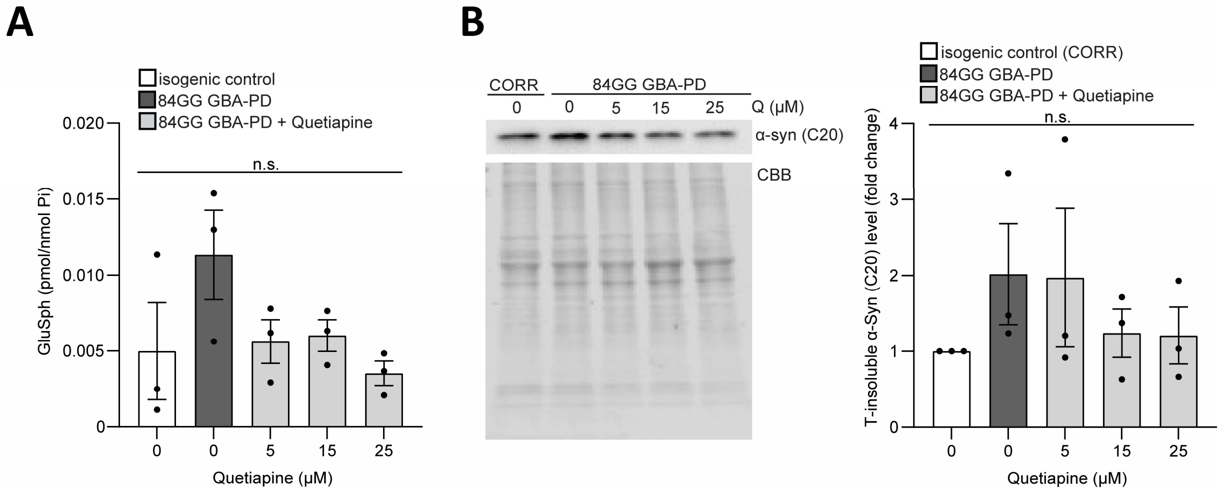

Figure S1. Quetiapine increases wild-type glucocerebrosidase and lowers pathogenic phenotypes in *GBA1*-linked iPSC-derived dopaminergic neurons. Heterozygous 84GG *GBA1* mutant dopaminergic neurons (84GG GBA-PD) and isogenic control neurons with *GBA1* mutation corrected by CRISPR-Cas9 gene editing (CORR) were treated with DMSO (vehicle) or quetiapine (5, 15, and 25µM) for 10 days. All samples were collected at day 130 of differentiation. **(A)** Triton-soluble lysates were analyzed for intracellular glucosylsphingosine (GluSph) by mass spectrometry normalized to internal phosphate (Pi) (N=3 independent experiments). **(B)** Immunoblot analysis of α-synuclein in Triton-insoluble lysates (N=3 independent experiments). Error bars, mean ± SEM. Q = Quetiapine. n.s. = not significant.

Figure S2

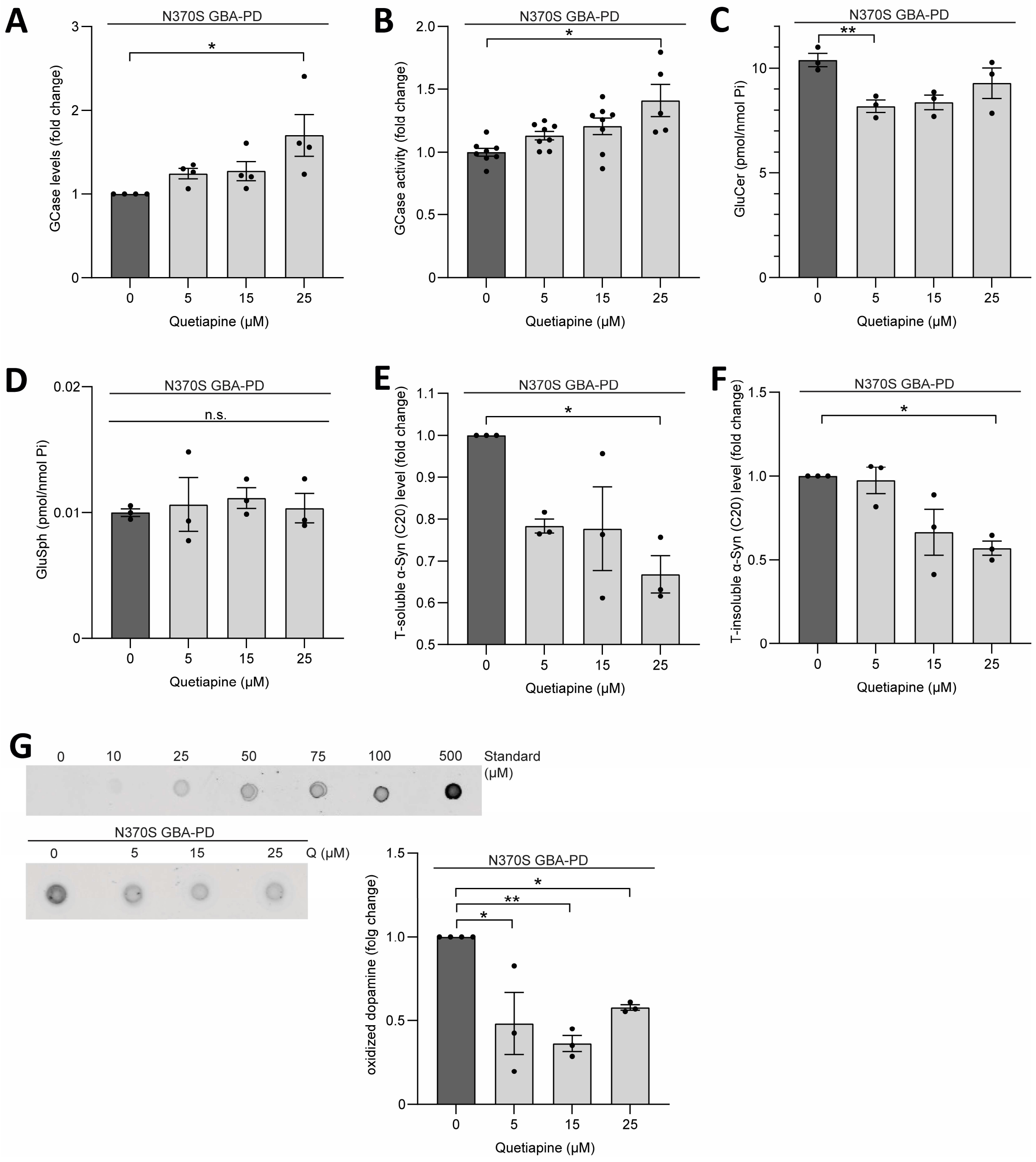

Figure S2. Quetiapine increases wild-type glucocerebrosidase and lowers pathogenic phenotypes in N370S *GBA1*-linked iPSC-derived dopaminergic neurons. Heterozygous N370S *GBA1* mutant dopaminergic neurons were treated with DMSO (vehicle) or quetiapine (5, 15, and 25μM) for 10 days. All samples were collected at day 70 of differentiation. Triton-soluble lysates were analyzed for **(A)** GCase protein by immunoblotting (N=4 independent experiments) and **(B)** GCase activity by *in vitro* enzyme activity assay (N=5-8 independent experiments). **(C)**

Quantification of intracellular total glucosylceramide (GluCer) species by mass spectrometry normalized to internal phosphate (Pi) (N=3 independent experiments). **(D)** Quantification of intracellular glucosylsphingosine (GluSph) by mass spectrometry normalized to internal phosphate (Pi) (N=3 independent experiments). **(E)** Immunoblot analysis of α-synuclein in Triton-soluble lysates (N=3 independent experiments). **(F)** Immunoblot analysis of α-synuclein in Triton-insoluble lysates (N=3 independent experiments) **(G)** Detection and quantification of oxidized dopamine (DA) performed by near-infrared fluorescence assay (N=3-4 independent experiments). Standard of oxidized DA ranging from 0 to 500μM shown. Error bars, mean ± SEM. *P<0.05 and **P<0.01, one-way ANOVA with Tukey post hoc test. Q = Quetiapine. n.s. = not significant.

Figure S3

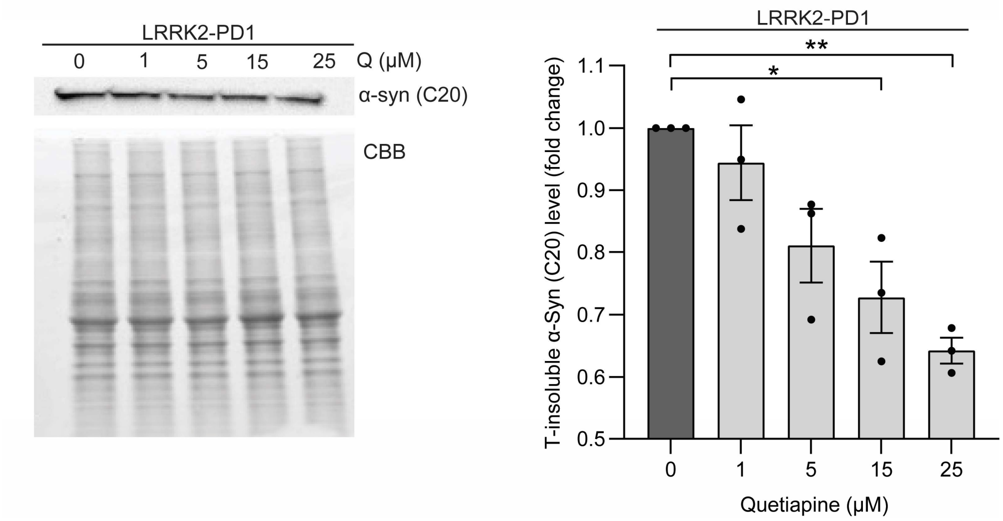

Figure S3. Quetiapine treatment leads to reduction of Triton-insoluble α-synuclein in LRRK2-linked PD iPSC-derived dopaminergic neurons. LRRK2 G2019S mutant dopaminergic neurons (LRRK2-PD1) were treated with DMSO (vehicle) or quetiapine (1, 5, 15, and 25μM) for 10 days. All samples were collected at day 100 of differentiation. Triton-insoluble lysates were analyzed for levels of α-synuclein (N=3 independent experiments). Error bars, mean ± SEM. $^{**}P<0.01$, one-way ANOVA with Tukey post hoc test. CBB = Coomassie Brilliant Blue. Q = Quetiapine.

Figure S4

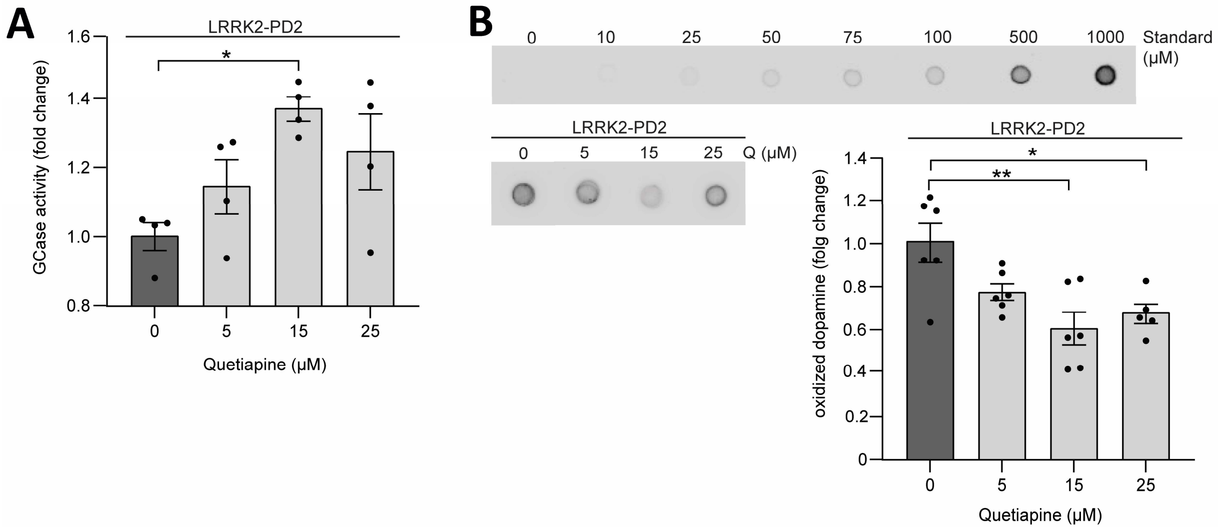

Figure S4. Quetiapine treatment leads to wild-type glucocerebrosidase activation and partial rescue of oxidized dopamine accumulation in LRRK2-linked PD iPSC-derived dopaminergic neurons. LRRK2 G2019S mutant dopaminergic neurons (LRRK2-PD2) were treated with DMSO (vehicle) or quetiapine (5, 15, and 25μM) for 10 days. All samples were collected at day 100 of differentiation. **(A)** Triton-soluble lysates were analyzed for GCase activity by *in vitro* enzyme activity assay (N=4 independent experiments). **(B)** Cell lysates were analyzed for oxidized dopamine (DA) by near-infrared fluorescence assay (N=5-6 independent experiments). Standard of oxidized DA ranging from 0 to 1000μM shown. Error bars, mean ± SEM. *$P<0.05$ and **$P<0.01$, one-way ANOVA with Tukey post hoc test.

Figure S5

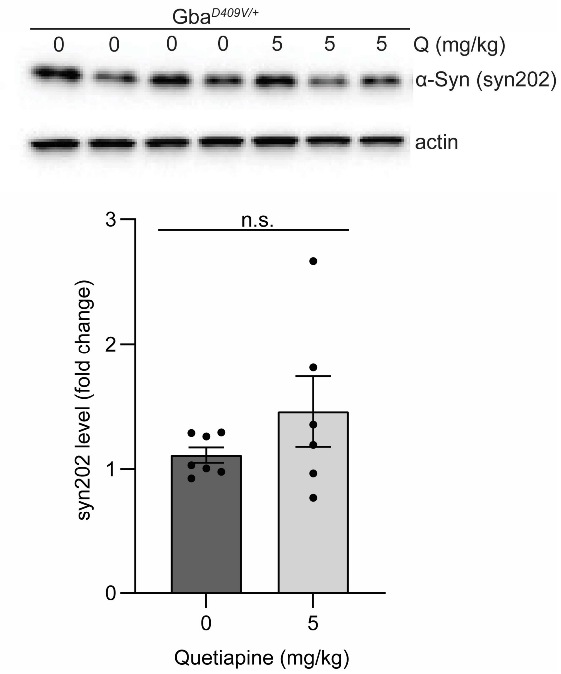

Figure S5. Wild-type glucocerebrosidase (GCase) activation by quetiapine treatment in mice does not affect Triton-soluble α-synuclein levels. Gba1$^{D409V/+}$ mutant mice were treated with saline (vehicle) or quetiapine (5mg/kg) intraperitoneally twice daily for 15 days. Immunoblot analysis of α-synuclein in Triton-soluble lysates of hippocampal tissue (n=7 saline and n=6 quetiapine-treated mice). Actin was used as loading control. n.s. = not significant.
